# A critical examination of sport discipline typology: identifying inherent limitations and deficiencies in contemporary classification systems

**DOI:** 10.3389/fphys.2024.1389844

**Published:** 2024-07-10

**Authors:** Magdalena Johanna Konopka, Hans Keizer, Gerard Rietjens, Maurice Petrus Zeegers, Billy Sperlich

**Affiliations:** ^1^ Department of Epidemiology, Maastricht University, Maastricht, Netherlands; ^2^ Institute for Healthcare Management and Health Sciences, University of Bayreuth, Bayreuth, Germany; ^3^ Human Physiology and Sports Physiotherapy Research Group, Vrije Universiteit Brussel, Brussels, Belgium; ^4^ MPB Holding, Heerlen, Netherlands; ^5^ Integrative and Experimental Exercise Science and Training, Institute of Sport Science, University of Würzburg, Würzburg, Germany

**Keywords:** athlete, classification, endurance, framework, strength, typologies

## Abstract

Exercise scientists (especially in the field of biomolecular research) frequently classify athletic cohorts into categories such as *endurance*, *strength*, or *mixed*, and create a practical framework for studying diverse athletic populations between seemingly similar groups. It is crucial to recognize the limitations and complexities of these classifications, as they may oversimplify the multidimensional characteristics of each sport. If so, the validity of studies dealing with such approaches may become compromised and the comparability across different studies challenging or impossible. This perspective critically examines and highlights the issues associated with current sports typologies, critiques existing sports classification systems, and emphasizes the imperative for a universally accepted classification model to enhance the quality of biomolecular research of sports in the future.

## 1 Introduction

Biomolecular research in sports delves into the molecular intricacies of exercise, encompassing disciplines like transcriptomics, epigenomics, proteomics, metabolomics, or genomics, also known as *sportomics*. (Sellami et al., 2021; Elrayess et al., 2022). The translation of biomolecular information such as genetics to observable physical traits in sports is a multifaceted process influenced by the interplay between genetic factors (Sellami et al., 2021; Ahmetov et al., 2022; Kim et al., 2022) and environmental influences. (Midgley et al., 2007; Hawley, 2013). Many biological determinants of training and adaption exist, such as immunological markers, (Hacker et al., 2023), lipid metabolism, (Lee et al., 2017), myokines, (Leuchtmann et al., 2021), or gut microbiota, (Nolte et al., 2023), among many others, (Holloszy and Booth, 1976; Joyner and Coyle, 2008; Beattie et al., 2014; Beiter et al., 2024), which contribute to the interindividual variability of exercise adaptation. Further, athletes belonging to different sports disciplines exhibit distinct characteristics encompassing morphological, physiological, and psychological aspects. (Araújo and Scharhag, 2016). It is essential for research designs in biomedical sports studies to focus on homogeneous groups of athletes to maintain appropriate levels of both internal and external validity. (Slack and Draugalis, 2001; Halperin et al., 2015; Lightfoot et al., 2021; Konopka et al., 2022). In general, internal validity refers to the integrity of the study’s design, ensuring that the findings accurately reflect the effects of the variables tested. External validity, on the other hand, concerns how well the results of a study can be generalized beyond the specific sample studied. Consequently, homogeneous groups of athletes will increase internal validity by controlling for confounding variables.

Although homogeneous groups are generally achievable only in animal research, the inclusion of diverse study populations has emerged as a notable limitation in this field, resulting in studies with low reproducibility. (Williams et al., 2014; Tanisawa et al., 2020). The clustering of athletes into dichotomous categories such as *endurance* or *strength* without referring to classification systems (Rankinen et al., 2016; Al-Khelaifi et al., 2020; Wojciechowicz et al., 2021; Ipekoglu et al., 2022) poses a methodological problem. Many *endurance* athletes include significant amounts of *strength* components into their training process. (Beattie et al., 2014). For instance, labelling canoeing as endurance sports (as suggested by current classification systems) (Voisin et al., 2014; Guilherme et al., 2018; Wojciechowicz et al., 2021) when this sport massively involves strength training (García-Pallarés and Izquierdo, 2011) hampers the investigation of the biomolecular components specific to aerobic sports. Similar classification issues arise in sports such as middle-distance running, rowing, cross-country skiing, and swimming, which also incorporate various strength training techniques, thereby necessitating both high aerobic capacity and muscle strength. Thus, broad classification represents a major limitation e.g., for meta-analyses, leading to non-significant findings or false negatives. In summary, indiscriminate clustering of athletes from diverse disciplines may constitute a significant limitation of biomolecular research in sports, necessitating a discussion on the various typologies in sports.

This perspective aims to achieve three objectives: 1) raise awareness about this issue, 2) provide a critical evaluation of the existing classification systems, and 3) initiate a call for action for future research endeavors.

## 2 Typologies in sports

In general, sport is a social construct defined by theories and models, as well as through various types and typologies (Krüger et al., 2022). Unlike theories and models, which aim to mirror realities, types and typologies help organize and structure social issues. Typologies simplistically condense information by categorizing similar characteristics and properties into a specific type, thereby reducing complexity. The aim is to maximize similarity within each type while maintaining the greatest possible diversity between the types. (Krüger et al., 2022).

In sports, various typologies exist and are often based on i) *performance level* such as national, international, amateur, high level, etc. or ii) *sports disciplines* encompassing more distinct physiological categories related to e.g., endurance, aerobic metabolism, neuro-muscular strength and power components, sprint, or mixed skills. Recently, McKay and colleagues presented a comprehensive framework that offers a standardized approach to classifying individuals’ performance levels (McKay et al., 2022). This framework utilizes training volume and performance metrics to assign individuals to specific tiers, including Tier 0: Sedentary, Tier 1: Recreationally Active, Tier 2: Trained/Developmental, Tier 3: Highly Trained/National Level, Tier 4: Elite/International Level, or Tier 5: World Class. By implementing this classification framework, exercise scientists can apply this classification prospectively during participant recruitment as well as use it in systematic reviews and meta-analyses retrospectively, thereby creating comparable study populations, particularly concerning athletes’ performance levels.

Another frequently utilized typology in sports is the categorization into *endurance* or *strength*, representing the two distinct physiological *extremes* of human adaptation to physical activity. Endurance training generally encompasses exercise durations of several minutes up to several hours at various exercise intensities, increasing the capacity to sustain repetitive high-intensity, relatively low-muscle contraction. Elevated endurance performance is linked to the ability to take up, transport and utilize oxygen to generate energy in form of adenosine triphosphate (ATP) via oxidative metabolism without necessary improvements in maximum muscle strength. (Holloszy and Booth, 1976). Thus, it is without doubt that the maximal oxygen uptake (VO_2max_) is a major determining factor for success in endurance sports. (Joyner and Coyle, 2008; Lorenz et al., 2013). Nevertheless, as the distance to be covered decreases, the anaerobic system and neuro-muscular strength of the active muscles becomes more important. (Ingham et al., 2008; Jones et al., 2010; Lorenz et al., 2013). Hence, the economy of movement, defined as the energy cost per unit distance, emerges as another critical physiological determinant in endurance sports. (Di Prampero et al., 1993; Ingham et al., 2008; Joyner and Coyle, 2008).

Strength training, in contrast, encompasses short-duration activity at high or maximal muscle contraction improving the ability to perform muscle contractions with high-tension of a single or relatively few repetitions (Suchomel et al., 2018). This type of ability is evident in Olympic weightlifting, powerlifting, and e.g., throwing events in track and field. Improved strength-related performance is accomplished through inter- and intra-neuromuscular motor learning, increased fiber-recruitment synchronicity, muscle cell hypertrophy, and, possibly, hyperplasia without changes in VO_2max_ or ATP generation via oxidative metabolism (McDonagh and Davies, 1984; Sale, 1988). In many team sports such as soccer, basketball or field hockey, the contribution and fraction of the aerobic and anaerobic systems as well as muscular power vary considerably and a clear distinction to a classification of either endurance- or strength-related sports is not possible.

Particularly in sports genetic research, athletes from different sports disciplines are often clustered into groups to increase the sample size of athletes. (Konopka et al., 2022). Within sports genetic literature *endurance athletes* are labelled based on: 1) the duration of exertion (e.g., >5 min (Wojciechowicz et al., 2021); >8 min (Peplonska et al., 2019); >10 min (Guilherme et al., 2019); >20 min (Nazarov et al., 2001); >30 min (Maciejewska-Karlowska et al., 2014)), 2) different distances (e.g., running ≥800 m (Kothari et al., 2011; Kothari et al., 2012); ≥1500 m (Ben-Zaken et al., 2013; Voisin et al., 2014; Papadimitriou et al., 2018); ≥3000 m (Papadimitriou et al., 2008; Scott et al., 2009; Yang et al., 2017); ≥5000 m (Tobina et al., 2010; Ash et al., 2011; He et al., 2011; Yvert et al., 2012); or ≥10000 m (Eynon et al., 2009a; Eynon et al., 2009b)), 3) or the Mitchell classification of sports. (Boraita et al., 2010; Di Girolamo et al., 2017; Al-Khelaifi et al., 2019; Al-Khelaifi et al., 2020; Claessen et al., 2020; Oudjedi et al., 2023). According to the Mitchell classification, a genome wide association study described following disciplines as *endurance*: biathletes, cross-country skiers, 800 + *m* runners, rowers, kayakers, canoers, speed skaters, short-trackers, swimmers, cyclists, race walkers, boxers, badminton players, basketball players, water polo players, football players, and ice hockey players. (Al-Khelaifi et al., 2020). Some articles classified ≥200 m swimmers as *endurance* (Mikami et al., 2011; Mikami et al., 2014) whereas one study classified 200–400 m swimmers as *sprint athletes*. (Chiu et al., 2011). Finally, various team sports (e.g., basketball, football, handball, etc.) have been labelled as *endurance* or *strength* sports, depending on the author group. (Turgut et al., 2004; Guilherme et al., 2022).

Noteworthy, in different research fields, such as motor control or neuroscience, other classification techniques are employed (e.g., open and closed skills exercise). (Gu et al., 2019). Consequently, classification frameworks in sports strongly depend on the specific research area and the questions under investigation.

**FIGURE 1:**
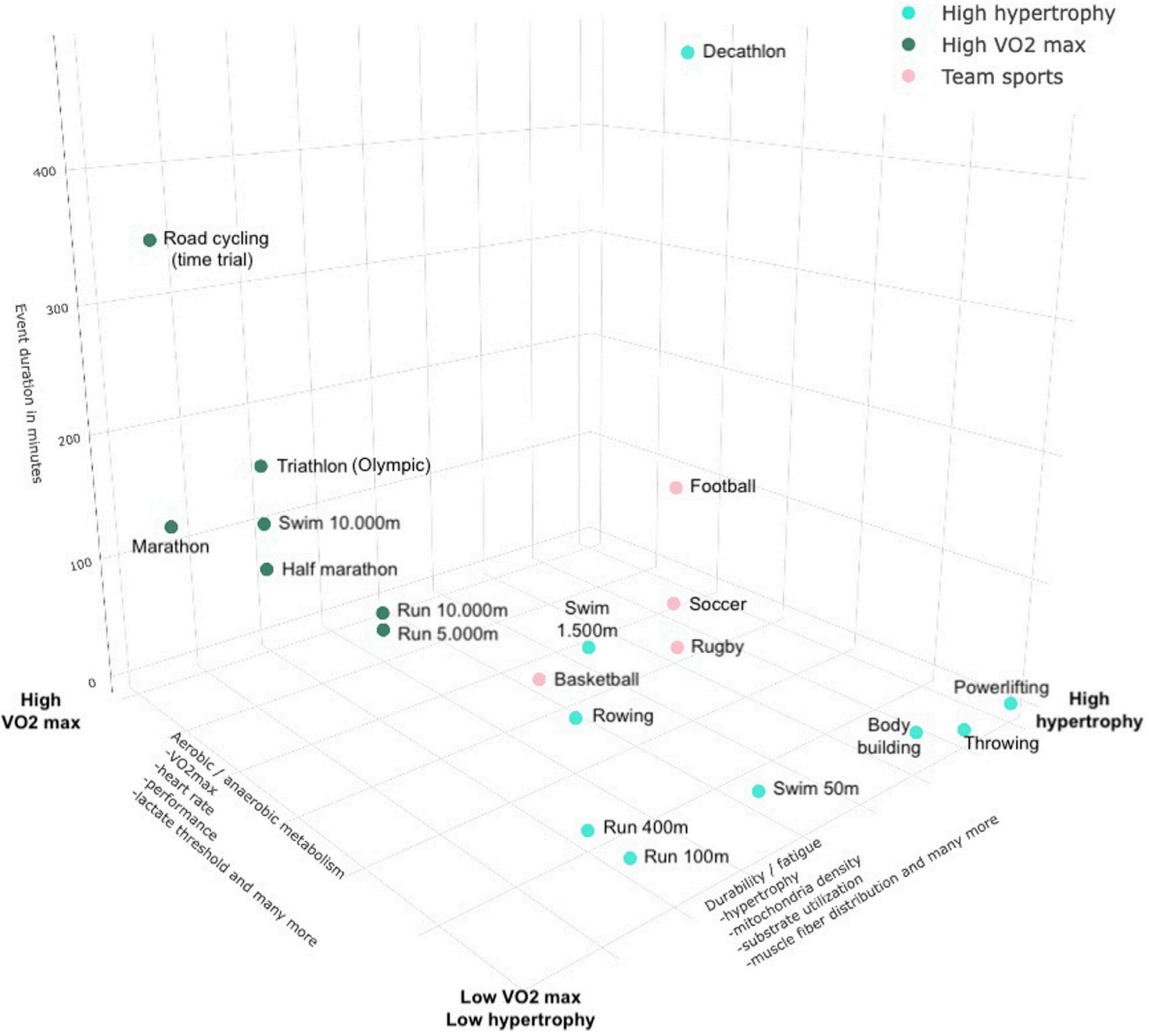
Key properties to consider in future classification approaches using continuous outcome data from lab and/or wearables.

In summary, the classification of sports into the dichotomous categories of *endurance* and *strength* has been widely employed; however, the diverse usage of these typologies prohibits the comparability of study populations and findings across different studies. Figure 1 depicts a three-dimensional representation of various sports disciplines.

This figure illustrates why dichotomous clustering of sports disciplines into categories like endurance or strength is problematic, as it is rare for a sport to be positioned at the extremes. Thus, the typologies in sports encompass several issues that need to be considered, taking into account various factors, which will be described in the following sections (Sperlich et al., 2023).

## 3 Issues about the *endurance* typology

One straightforward approach to categorizing athletes into *endurance* is by using their respective sports disciplines (e.g., swimming, running, soccer, etc.). However, it is crucial to also consider the time component of each exercise, which varies significantly not only between different sports disciplines but also within the same discipline (e.g., 50 m sprint swimming vs. long-distance swimming e.g., 10 km open-water swimming). Additionally, the major muscle groups involved (e.g., lower-body, upper-body, or whole-body), the proportion of fast and slow twitch muscle fibers, and the type of muscle contraction employed (concentric, concentric-eccentric) exhibit variation across different sports and must be considered when labelling *endurance* athletes. Furthermore, the balance between *specific*, *semi-specific* (e.g., training on a kayak ergometer for kayakers), and *non-specific training* (e.g., cycling for speed skaters) represents a critical factor in classifying endurance capabilities. (Sperlich et al., 2023). Lastly, the predominant type of energy metabolism utilized (fat/carbohydrates) holds significance and warrants consideration. Undoubtedly, numerous other factors (predominant ambient conditions including influence of altitude, heat and cold exposure) discriminate different sports disciplines and should be addressed when discussing typologies related to endurance in sports.

## 4 Issues about the *strength* typology

The typology of *strength* presents several noteworthy issues. Depending on the sports, strength training may encompass different objectives including maximal voluntary strength, explosive power, hypertrophy, or muscular endurance. (Stone et al., 2022). The lack of consensus of the specific traits encompassed by the strength typology poses challenges in precisely defining and studying this category. As an example, the intensity and number of repetitions as well as contraction forms (eccentric vs. concentric vs. isometric contraction) may target more neuro-muscular adaptation often evident in greater maximum voluntary force, rate of force development and improved stretch-shortening cycle without significant increase in muscle mass, (Douglas et al., 2017), whereas strength training could also target distinct stimuli inducing pronounced skeletal muscle hypertrophy. (Joanisse et al., 2020). There is a need to consider the influence of various traits, such as those related to muscle fiber type, neuromuscular coordination, or muscle architecture, which collectively contribute to an individual’s strength capabilities. (Suchomel et al., 2018). Moreover, the multidimensionality of strength-related traits requires comprehensive assessments beyond isolated measures, necessitating a holistic approach to understand the biomolecular underpinnings of strength. Therefore, refining and standardizing the strength typology is essential for meaningful and rigorous research in this area. Thus, there are various factors to consider when referring to the term *endurance* or *strength*.

## 5 Current classification approaches and their shortcomings

In 1999, an initial endeavor was made to establish a *taxonomy of sports* rating system. (Stefani, 1999). In this rating system, sports disciplines were classified as either: i) *combat sports* where competitors aim to control the opponent, ii) *object sports* where competitors aim to control an object in direct competition with the opponent, or iii) *independent sports* where competitors are not impeded by opponents. However, to the best of our knowledge this taxonomy was never applied in biomolecular research of sports, rightly so.

Next, the Mitchell Classification takes into account static and dynamic components when categorizing sports (Mitchell et al., 2005). Static sports involve the generation of significant intramuscular forces with minimal or no change in muscle length, typically assessed through measures such as maximal voluntary contraction. These sports are often associated with muscular strength and therefore are commonly labelled as *strength* disciplines. Conversely, dynamic sports involve changes in muscle length and joint movement with relatively lower intramuscular forces. In these types of exercises, the VO_2max_ assumes greater importance as a determining factor for performance (Mitchell et al., 2005; Sanz de la Garza et al., 2020). Accordingly, these sports are often referred to as *endurance* disciplines. Another classification approach, similar to the Mitchell classification, is based on isotonic (changes in muscle length) and isometric (no changes in muscle length) components (Niebauer et al., 2018; Sanz de la Garza et al., 2020). It is essential to note that these classification systems were originally designed to assess the risk and safety of athletes with cardiovascular abnormalities, thus they are not suitable for categorizing healthy athletes. In both classification systems, sports such as boxing, badminton, soccer, and middle-distance running are labelled as endurance sports, disregarding the numerous factors mentioned earlier (e.g., underlying muscle groups, time components, energy metabolism, etc.). In conclusion, these two approaches represent simplistic methods of categorization and should not be applied in the field of biomolecular research. (Al-Khelaifi et al., 2019).

In light of the multifaceted nature of numerous sports disciplines, which necessitate a synergistic blend of strength and endurance training to achieve optimal performance, there has been a recent development of a hypothetical model known as the *strength-endurance continuum* (Nader, 2006). This model aims to capture the continuum between these two key components of athletic performance and recognizes that many sports, including middle distance running, rugby, football, and swimming, among others, rely on a balanced integration of both strength and endurance capacities (Nader, 2006). This model considers several factors described above, but still does not ensure creating homogenous athlete groups.

The utilization of current classification systems in the categorization of athletes leads to a lack of internal validity in the findings of biomolecular research in sports. It is imperative to avoid treating diverse groups such as long-distance runners and boxers as a single entity, as a recent genome-wide association study did (Al-Khelaifi et al., 2020). Furthermore, it is problematic when meta-analyses omit reference to any classification system altogether, as observed in a recent meta-analysis (Ipekoglu et al., 2022). Unfortunately, numerous scientific articles within this research field fail to adequately describe the athletic cohorts and sports disciplines under investigation, neglecting essential details such as distances, time, and performance levels, yet still assign them to labels such as *endurance* or *strength* athletes.

## 6 Further aspects to consider and call for action

In sports science, it is well recognized that an elite performance status involves the interaction between multiple biological and environmental factors (Myburgh, 2003). Research into the biological determinants and adaptions to strength and/or endurance training has identified various biomarkers (Lee et al., 2017; Leuchtmann et al., 2021; Lavin et al., 2022; Hacker et al., 2023; Nolte et al., 2023) such as hormones, enzymes, cytokines, myokines, etc. offering similar characteristics and properties to cluster on.

Noteworthy,athletes also exhibit significant variations in their off-training behavior (Exel et al., 2018) which can have a profound impact on biomolecular analyses and therefore warrants careful consideration. Researching continuous outcome data rather than categories of sports may offer a solution and can be derived from lab-analyses or self-measurements. Wearable technology offers unique opportunities for data collection and analysis of various modulators of adaptation including sleep, free-time activity, diet, emotions, etc. (Vijayan et al., 2021) Athletes usually wear these devices during training and competition, enabling continuous monitoring of factors like heart rate, sleep patterns, energy expenditure. (Moore and Willy, 2019). Wearable data can be invaluable for understanding the biomolecular underpinnings of athletic performance and identifying key factors that contribute to success in specific sports disciplines (McDevitt et al., 2022). This type of data would allow to retrieve information about the time and volume of concentric, eccentric, and isometric muscle contraction, the number of jumps, sprints, and time spent in different exercise intensity zones (Lutz et al., 2019). The clustering of athletes based on such continuous data rather than sports disciplines enables the grouping of similar physiological characteristics independent of sport discipline or player position and may lead to new findings in the future.

Finally, we recognize that creating fully homogeneous groups of athletes is unfeasible due to numerous confounding factors. Nonetheless, there are certain factors that should guide the classification of athletes’ profiles rather than the classification of sports disciplines. Therefore, we aim to stimulate further discussion and research on this topic and to encourage a reevaluation of the continued use of dichotomous athlete classifications.

To facilitate future research in the field, we suggest considering the following.

i. It is critical to consider the research question being addressed.
ii. Use continuous outcome data derived from lab-analyses or self-measurements. (Table 1 summarizes the main advantages and disadvantages of clustering either by sports disciplines or employing continuous data).
iii. It is advisable to cluster based on theoretical logic. For instance, it is less problematic to examine middle-distance and 3000 m runners as they share comparable physiological (biological) characteristics as a result of inherited traits, training strategies and competitions. However, comparing a 50 m swimmer to a 1500 m swimmer may pose challenges due to their substantial differing physical demands.

**TABLE 1:** (Dis)advantages of both approaches: sport disciplines vs. continuous outcome data.

Classification based on	Sport disciplines (endurance vs. strength)	Continuous outcome data (from labs or wearables)
Advantages	- Easy to cluster - Convenient to apply - Large grouping possible	- Objectively measured - Easy to measure - Data availability and data sharing - Large datasets available - High internal validity - High generalizability across studies - Meta-research possible
Disadvantages	- Oversimplification and subjectivity of clustering - Low internal validity - Low generalizability across studies	- Low validity of self-measurements - Complex data management (big data) - Ethical concerns may arise, such as privacy and third-party access

We believe that considering these suggestions will provide a new approach for researching Nonetheless regardless of the classification approach adopted, whether focusing on disciplines or profiles, typologies inherently will always contain some degree of information reduction (Krüger et al., 2022).

## 7 Conclusion

The issue regarding the typologies of athlete groups poses a significant challenge in comparing the results of studies focusing on the biomolecular pathways underlying *endurance* or *strength* sports. To address this issue and enhance the field of sportomics it seems crucial to establish an accepted classification system for categorizing sports disciplines among healthy athletes in the future. Such a system would offer several advantages, including increased internal validity, the ability to generate comparable results across studies, and the potential for uncovering novel findings. Conducting large-scale meta-analyses with high-quality data becomes feasible with a standardized classification system, which is essential for advancing research in this field. By stimulating further discussion and collaboration on this important topic we aim future advancements within biomolecular research of sports.
